# Calcium in Red Blood Cells—A Perilous Balance

**DOI:** 10.3390/ijms14059848

**Published:** 2013-05-08

**Authors:** Anna Bogdanova, Asya Makhro, Jue Wang, Peter Lipp, Lars Kaestner

**Affiliations:** 1 Institute of Veterinary Physiology, Vetsuisse Faculty and the Zürich, Center for Integrative Human Physiology, University of Zürich, Zürich 8057, Switzerland; E-Mails: annab@access.uzh.ch (A.B.); makhro@vetphys.uzh.ch (A.M.); 2 Institute for Molecular Cell Biology and Research Centre for Molecular Imaging and Screening, Saarland University, Homburg/Saar 66421, Germany; E-Mails: jue.wang@uniklinikum-saarland.de (J.W.); peter.lipp@uks.eu (P.L.)

**Keywords:** erythrocyte, senescence, clot formation, thrombosis, cytoskeleton

## Abstract

Ca^2+^ is a universal signalling molecule involved in regulating cell cycle and fate, metabolism and structural integrity, motility and volume. Like other cells, red blood cells (RBCs) rely on Ca^2+^ dependent signalling during differentiation from precursor cells. Intracellular Ca^2+^ levels in the circulating human RBCs take part not only in controlling biophysical properties such as membrane composition, volume and rheological properties, but also physiological parameters such as metabolic activity, redox state and cell clearance. Extremely low basal permeability of the human RBC membrane to Ca^2+^ and a powerful Ca^2+^ pump maintains intracellular free Ca^2+^ levels between 30 and 60 nM, whereas blood plasma Ca^2+^ is approximately 1.8 mM. Thus, activation of Ca^2+^ uptake has an impressive impact on multiple processes in the cells rendering Ca^2+^ a master regulator in RBCs. Malfunction of Ca^2+^ transporters in human RBCs leads to excessive accumulation of Ca^2+^ within the cells. This is associated with a number of pathological states including sickle cell disease, thalassemia, phosphofructokinase deficiency and other forms of hereditary anaemia. Continuous progress in unravelling the molecular nature of Ca^2+^ transport pathways allows harnessing Ca^2+^ uptake, avoiding premature RBC clearance and thrombotic complications. This review summarizes our current knowledge of Ca^2+^ signalling in RBCs emphasizing the importance of this inorganic cation in RBC function and survival.

## 1. Introduction

Ca^2+^ is a universal and ubiquitous signalling molecule [1,2], regulating cell cycle and fate, metabolism and structural integrity, motility and volume. Most of the Ca^2+^ in the cytosol is bound and buffered by numerous Ca^2+^ binding proteins, phospholipids and inorganic phosphate. When bound and buffered Ca^2+^ are included, total intracellular Ca^2+^ in red blood cells (RBCs) reaches 5.7 μM [3]. Basal free Ca^2+^ concentration in RBCs of healthy human beings under physiological conditions is estimated to be in the range of 30 to 60 nM [4]. A tremendous gradient of at least 40,000-fold between the cytosol and blood plasma where free Ca^2+^ concentration reaches up to 1.8 mM is maintained due to particularly low permeability of membranes to Ca^2+^ (~50 μmol/(l^cells^ h)) and efficient extrusion of Ca^2+^ from the cells by the plasma membrane Ca^2+^ pump (PMCA) [4]. This gradient may be used for signalling purposes as opening of a few hundreds of channels transporting 10^6^ ions per second over several milliseconds may result in >10-fold changes in free Ca^2+^ levels in the sub-membrane space, causing acute changes in the activity of multiple Ca^2+^ sensitive proteins involved in structural, signalling, metabolic and transport functions. Such a huge Ca^2+^ influx seems to exceed the RBC’s Ca^2+^-buffering abilities. Long-term increases in the Ca^2+^ permeability result in serious dysregulation of multiple cellular functions. The subsequent onset of proteolysis, oxidation, irreversible shrinkage and phosphatydylserine (PS) exposure to the extracellular membrane leaflet facilitate clearance of Ca^2+^ overloaded cells in case the latter have not been haemolysed when passing through capillaries.

This review summarizes the current knowledge on the regulation of RBC Ca^2+^ levels, Ca^2+^ dependent processes and the potential role that Ca^2+^ might play in the development of RBC pathologies. For obvious reasons this review makes no claim to be complete, but wants to draw attention to open questions and mechanisms to be resolved in the field of Ca^2+^ signalling in RBCs.

## 2. Ca^2+^ Transport across the RBC Membrane

### 2.1. Ca^2+^ Extrusion Pathway: Plasma Membrane Ca^2+^ Pump

It was realised very early on that the inhibition of the energy supply in RBCs leads to a Ca^2+^ increase [5]. Although the nature of the Ca^2+^ influx remained unknown for several decades, the extrusion mechanism was realized to be mediated by a plasma membrane Ca^2+^ pump (PMCA). For human RBC membrane the presence of the B-splice isoform of the PMCA1 was shown [6]. This P-type ATPase is ubiquitously expressed. It is composed of 1220 amino acids forming ten transmembrane domains, two intracellular loops containing ATP binding and phosphorylation sites and inward-facing *N*- and *C*-terminals. The latter contains a Ca^2+^ calmodulin binding domain, phosphorylation sites and a PDZ-binding domain serving as a docking terminal for a number of proteins [7]. The maximal Ca^2+^ extrusion rate in RBCs can vary within RBC populations of a single donor between >60 mmol/(l_cells_ h) and <4 mmol/(l_cells_ h) [8]. The maximal turnover rate of the PCMA is significantly higher in RBCs from light fractions compared to that in RBCs within dehydrated RBC fractions following density centrifugation [9]. This reduction in *V*_max_ in cells from the dense fraction was referred to as a hallmark of RBC senescence [10]. The maximal Ca^2+^ turnover rate of PMCA in the RBC membrane of healthy humans not loaded with Ca^2+^ is most likely never reached. The apparent *V*_max_ for PMCA measured in such cells is ~50 μmol/(l_cells_ h), the value equal to the passive Ca^2+^ uptake. The enzyme half-activation constant for Ca^2+^ was reported to be 4 μM, far above the actual free cytosolic Ca^2+^ levels in human RBCs [11].

Increases in the intracellular free Ca^2+^ are sensed by the PMCA and occur in response to the interaction of the Ca^2+^ calmodulin complex with the *C*-terminus of the enzyme. In Ca^2+^-loaded RBCs the limiting factor of the PMCA transport capacity is ATP availability. The pump is fuelled preferentially by a pre-membrane ATP pool [8,12,13] and has a *K*_d_ (ATP) of 145 μM [5,14]. Under conditions of permanent Ca^2+^ leak, activation of PMCA results in rapid ATP depletion.

RBC-derived plasma membrane vesicles contain factors activating PMCA, such as arachidonic acid, ceramide and acidic phospholipids, whereas sphingosine suppresses its function [15–17]. The function of the PMCA is extremely temperature-sensitive with ~30-fold reduction in turnover rate for every 10 °C drop [18].

### 2.2. Ca^2+^ Influx Pathways

Ca^2+^ influx through the plasma membrane of healthy human RBCs is extremely slow but increasing up-to 5-fold in cells of patients with sickle cell disease (SCD) and several other forms of hereditary haemolytic anaemia [4]. Several cation channels mediate inward movement of Ca^2+^. Significant progress in identification of ion channels in control of Ca^2+^ uptake has recently been reviewed [19].

Voltage-activated non-selective cation channels [20–22] were shown to be Ca^2+^ permeable [23]. These initial observations have all been performed on excised patches, but the presence of the channel was also shown in the whole cell configuration [24] and further flux-based characterisations have been performed [25–27]. However, a proof of the molecular identity remains to be provided.

Furthermore, a P-type Ca^2+^ channel was pharmacologically identified [28] and shown to be a Ca_V_2.1 channel by Western blot analysis [29]. This channel can be inhibited by ω-agatoxin TK [29]. However, in contrast to the initial investigations, in which activation by protein kinase C (PKC) was proposed, recent findings depicted a rather indirect interaction with PKC [30].

A recent report provided evidence for the presence of a transient receptor potential (TRP) channel of subtype C6 in the RBC membrane [31]. However, most of the work done so far was performed on murine RBCs and detailed characterization of this channel in human RBCs is missing.

In addition, the expression of an NMDA receptor channel was initially reported for rat [32] and later in human RBCs using molecular biological and electrophysiological approaches [33]. NMDA receptor agonists include glutamate, *N*-methyl d-aspartate (NMDA), homocysteine, homocysteic acid, glycine and d-serine [34].

Recently, the protein PIEZO1 was reported as being mutated in RBCs in hereditary xerocytosis [35] without knowing its physiological function. However, PIEZO1 is characterized as a mechano-sensitive cation channel in heterologous expression systems [36,37].

Furthermore there is evidence for an AMPA receptor related channel activity in RBCs [38].

All the channels mentioned above were reported to be present in human RBCs from healthy donors. However, some currents were only shown to be present in cells of patients. An example is an increase in non-selective cation conductance on RBC of SCD patients mediating or contributing to P*_sickl_*_e_ [39,40], an increased membrane permeability in SCD RBC. It is still not completely clear if this reflects an increased activity of one or more of the above mentioned channels or yet another conductance [40,41]. However, recent investigations provide evidence for the involvement of the NMDA receptor [42].

## 3. Ca^2+^-Sensitive Proteins in RBCs

### 3.1. Onset of Ca^2+^-Inducible Events and Ca^2+^ Sensors in RBCs

**Figure 1:**
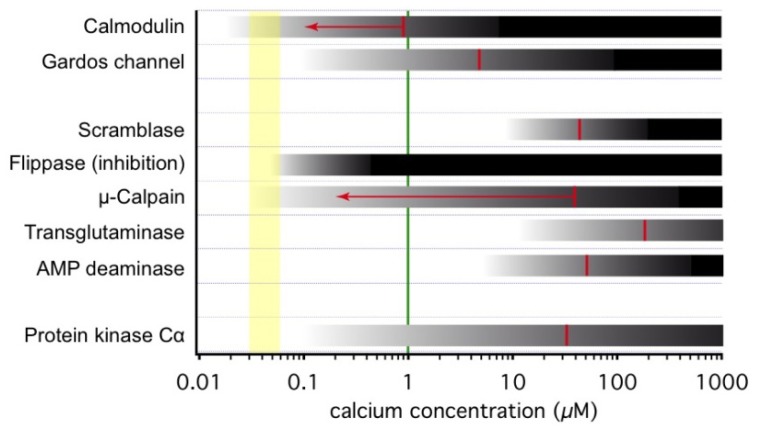
Overview of concentration dependence of Ca^2+^ activated events in RBCs. The yellow column indicates the estimated range of RBCs’ resting free Ca^2+^ [4]. The gray/black bars indicate the activation of the proteins with the intensity of darkness related to the activation level (details see below). The red lines depict the half activation concentration. For orientation, the green line provides the *in vivo k*_D_ for Fluo-4 [47], probably the most appropriate Ca^2+^ fluorophore to be used in RBCs [48]. The universal intermediate messenger calmodulin has a dissociation constant for Ca^2+^ of 920 nM [49], which can be shifted down to 100 nM (see main text), indicated by the red arrow. The Gardos channel has an open probability of EC_50_ of 4.7 μM with a Hill slope factor of approximately 1 [50]. Values were measured in excised patches at a membrane potential of 0 mV. The curve of the opening frequency is almost superimposable (EC_50_ of 4.3 μM) [50] keeping the values given in the figure valid also for whole cell and hence population based investigations. The values for half maximal activation of the scramblase were determined by different studies with varying methodologies and a slightly different result. Values varied between approximately 30 μM determined in liposomes [51] and 70 μM measured in RBC ghosts [52]. The flippase depicts almost full inhibition already at a Ca^2+^ concentration of 400 nM [53]. μ-Calpain, a protein that cleaves cytoskeleton and membrane proteins depicts half activation at 40 μM Ca^2+^ [54] but can be activated and then shifting half-maximal activation down to 200 nM [55]. Transglutaminase mediating polymerisation of RBC membrane proteins in its native form has a dissociation constant for Ca^2+^ of 190 μM [56]. Adenosine monophosphate (AMP) deaminase is an enzyme that converts AMP into inosin monophosphate and is directly stimulated by Ca^2+^ at a half maximal concentration of 50 μM free Ca^2+^ [57]. The binding of Ca^2+^ to the C2-domain of PKCα was determined *in vitro* to be 35 μM with a Hill coefficient of 0.9 [58]. Although the Ca^2+^ dependence of the membrane binding was measured to be an order of magnitude lower [58], the initial Ca^2+^ binding is the crucial step for PKCα activation and therefore the relevant number in this compilation.

**Figure 2:**
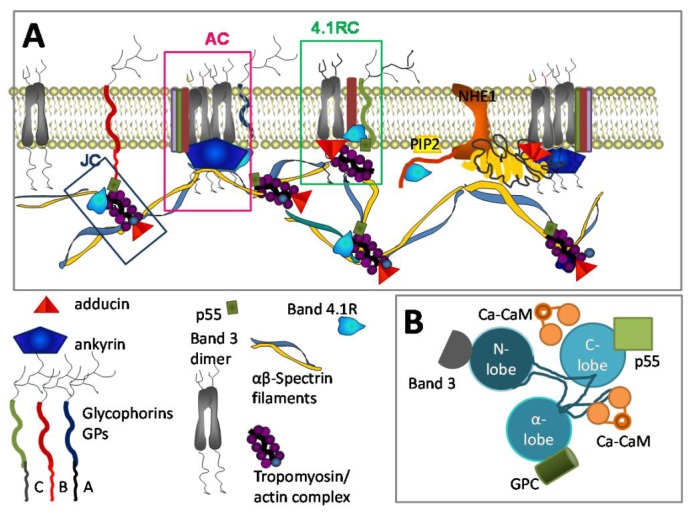
Ca^2+^ and RBC cytoskeleton. (**A**) Ca^2+^ sensitive elements of the cytoskeleton: band 4.1 and adducin interact with the Ca^2+^-calmodulin (Ca-CaM) complex. Adducin binds to actin blocking elongation of the fast-growing (barbing) ends of actin filaments within junctional complexes (J). When interacting with the band 3 dimers anchoring the spectrin network to the membrane, J become a part of bigger multi-protein complexes known as 4.1R-comples (4.1RC). Interaction with Ca-CaM down-regulates capping activity of adducin regulating thereby actin filament assembly [59]. Furthermore, adducin tetramers participate in docking of carbonic anhydrase II (CAII) to band 3 tetrames. NHE1 is activated as it joins CAII and thereby becomes associated with the ankyrin complex (AC) [60,61]. Band 4.1R is an interacting partner of a number of proteins. Those include spectrin and actin which bind to the 10 kDa domain of the band 4.1R protein; band 3 protein, p55, and GPC docking to the FERM domain of it and NHE1 interacting with its *C*-terminal 24 kDa domain. Interaction of band 4.1R with Ca-CaM triggers the reduction of the affinity of this protein to all interacting partners. As a result, spectrin network interaction with the integral proteins becomes loose. Decrease in affinity of band 4.1R to the cytosolic domain of NHE1 favours its dissociation from 4.1R and interaction with phoshatidylinositol 4,5-phosphate (PIP_2_), thus causing NHE activation [62]. PIP_2_ also modulates interaction of band 4.1R with glycophorin C and band 3 protein [63]; (**B**) Schematic representation of the FERM (4.1/ezrin/radixin/moesin) domain of band 4.1 protein, indicating docking ports for interacting partners and Ca-CaM binding sites (for details see [64]).

When in the cell, Ca^2+^ activates numerous Ca^2+^ dependent proteins. Each of them has its own activation threshold. Thus, gradual increase in Ca^2+^ levels is associated with gradual activation of various groups of Ca^2+^-sensitive proteins involved in physiological and pathophysiological processes in RBCs. In Figure 1 we compiled current knowledge about the activation ranges of some selected proteins. This list of Ca^2+^ sensitive proteins is by far not comprehensive and can hardly be covered within one review. Despite a large number of such proteins and diversity of their functions, only few of them are “true” Ca^2+^ sensors interacting directly with calcium ions [43]. One of such ubiquitous sensors highly abundant in RBCs is calmodulin. Calmodulins 1–4 (CaM) are 17 kDa proteins comprising two globular EF hand Ca^2+^ binding domains enriched with carboxyl and carbonyl groups (Asp, Glu and Thr) interconnected with a flexible linker (for details see, e.g., [44,45]). Upon interaction with Ca^2+^, CaM wraps around amphipathic regions of the protein compacting into a globular shape and pulling the interacting domains of the target out of lipophilic pockets or out of the membrane lipid bilayer moiety. In RBCs the proteins regulated by interaction with the Ca^2+^ calmodulin 2 complex (Ca-CaM) include, e.g., elements of the cytoskeletal network, the Na^+^/H^+^ exchanger NHE1, PMCA and the endothelial NO synthase (eNOS). Cytoplasmic CaM becomes active when recruited to the plasma membrane where its action is often coupled to that of phosphatidylinositol 4,5-bisphosphate (PIP_2_) localised at the inner leaflet of the membrane. An example of such coupling is a competitive binding of both co-regulators to the intracellular domain of NHE1 (see Figure 2A).

Another class of “true” Ca^2+^ sensors (~650 proteins included) contain Ca^2+^ binding C2 domains interacting with 2–3 Ca^2+^ [43,46]. In RBCs proteins with C2 domains include phospholipases, PKCα, phosphoinositide 3-kinase (PI3K) and many others. Binding of Ca^2+^ to the C2 domains, triggers translocation of these proteins to the specific areas within plasma membrane containing their substrates [46].

### 3.2. Ca^2+^-Dependent Phosphorylation

Changes in phosphorylation are among the most important modulations of protein activity in RBCs. Among the kinases there is a group of Ca^2+^ activated protein kinases, the conventional protein kinase C (cPKC) [65]. Among cPKCs, only protein kinase Cα (PKCα) can be found in RBCs [66]. Upon Ca^2+^ binding, PKCα translocates to the plasma membrane, where it phosphorylates its target proteins. The kinase domain of PKCs lacks specificity [67,68] and therefore numerous proteins can be phosphorylated. Reports include the PMCA [69], cytoskeletal proteins ([70,71] and see below), NADPH oxidase [72] and possibly further proteins [29,30,73] are affected.

### 3.3. Ca^2+^ and RBC Cytoskeleton

Opening of cation channels in response to mechanical stress and the presence of activators, such as amino acids, proinflammatory cytokines and others, result in local transient increase in Ca^2+^ levels in the vicinity of the plasma membrane. The latter, most likely serves as a signal to mediate rapid reversible changes in cytoskeletal flexibility.

The calcium-calmodulin complex (Ca-CaM) plays a key role in regulation of cytoskeletal stability. Selected elements of the cytoskeletal architecture interacting with Ca-CaM are schematically shown in Figure 2 together with their interacting partners. Those include major components of the cytoskeletal network, protein 4.1R, and adducin. These proteins function as docking stations for spectrin and actin, band 3 protein, glycophorins protein 4.2 and p55 and form a complex known as ankyrin-based complex and junctional complex (Figure 2A) [74,75]. The junctional complex is formed by the three principal components of the skeletal network junctions (spectin, actin, and 4.1R, together with tropomyosin, tropomodulin, adducin, dematin, p55). When associated with transmembrane proteins, GPC, XK, Kell, Duffy, band 3, and Rh junction complex forms a multiprotein 4.1R-based complex [76].

It is shown in Figure 2A, that the band 4.1R protein is a key constituent of all three complexes. Upon interaction with Ca-CaM, affinity of the 4.1R protein to all the interacting partners decreases and the cytoskeletal structure including spectrin-actin-tropomyosin junctions and the spectrin-actomyosin web interaction with the transmembrane protein clusters becomes loose and unstable. Such, Ca-CaM induced dissociation of NHE1 from the ankyrin complexes and band 4.1R protein. This facilitates the interaction of NHE1 with PIP_2_ resulting in activation of this ion transporter and dysregulation of cell volume and cytosolic alkalosis [62,77].

The Ca^2+^ concentration required for half-activation of calmodulin is 100 nM [78]–920 nM [49], Figure 1, thus no significant dissociation of the membrane cytoskeleton is expected to occur under physiological concentration of intracellular free Ca^2+^ [79]. Phosphorylation of calmodulin at Ser-80 and -84 affects its affinity to 4.1R reducing their binding [80].

Ca^2+^ dependent phosphorylation is a second mode of action of Ca^2+^ on cytoskeletal proteins. Phosphorylation of the 4.1R protein at serine 312 and serine 331 by PKC was reported [81]. These two phosphorylation sites are localised within a domain flanked by the spectrin- and actin binding domain and a domain containing the interaction sites for transmembrane proteins (Figure 2B). When phosphorylated the 4.1R-β-spectrin interaction appears to be weakened by ~30% [81]. As Ca^2+^ uptake is known to be triggered by mechanical deformations [82], controlled reversible loosening of cytoskeletal network in cells passing through capillaries is an advantage. Uncontrolled irreversible loss of cytoskeletal stability in RBCs of patients with haemolytic anaemia, in which Ca^2+^ is permanently upregulated, on the contrary compromises mechanical stability of the RBC membrane [83]. In RBCs of healthy humans the 4.1R protein is not phosphorylated [81]. One more target of PKC is α-adducin which, upon phosphorylation at Ser-726 decreases its affinity to F-actin, as it was observed in SCD RBCs. After the loss of F-actin capping dissociation of spectrin from actin occurs [84], which results in RBC inability to change shape.

In RBCs transaminase 2 responds to Ca^2+^ entry with a rapid release of its inhibitory GTP and activation resulting in formation of Nɛ (γ-glutaminyl)lysine cross-linking (Figure 1). As a result, in Ca^2+^ overloaded RBCs formation of numerous polymeric protein complexes such as Glut1-adducin-dematin adducts as well as cross-linked complexes of Band 3-ankyrin-spectrin and glycophorin *C*-band 4.1-p55 occurs [85]. This Ca^2+^ induced remodelling of the cytoskeletal structure and concomitant changes in cell shape and membrane plasticity are suggested to contribute to premature RBC clearance.

### 3.4. Ca^2+^ and RBC Volume Regulation

RBC volume regulation is a complex process with contributions of numerous molecular players including, e.g., band 3 protein [86]. A marked volume decrease is mediated by the so-called Gardos effect, which was among the first Ca^2+^ dependent processes recognized in RBCs [87]. The corresponding Gardos channel was the first channel measured by patch-clamp in RBCs [88,89]. This channel is a Ca^2+^ activated K^+^ channel and the Gardos effect represents the Ca^2+^ induced K^+^ loss of RBCs. The channel is characterized by a single channel conductance of approximately 20 pS [88], a selectivity of K^+^ to Na^+^ of about 15:1 [90] and an EC_50_ (Ca^2+^) of 4.7 μM, Figure 1 with a Hill-slope of approximately 1 [50]. Later, the molecular identity of the Gardos channel was shown to be the hSK4 channel [91]. Functionally, the opening of the Gardos channel leads to a hyperpolarisation and a loss of K^+^, Cl^−^ and water resulting in cell shrinkage. Although the physiological function of the Gardos channel is not completely elucidated, there are two complementary concepts: (i) Openings of RBC Ca^2+^ channels by platelet released substances [92,93] initialy trigger the consecutive activation of the Gardos channel. This Gardos channel mediated dehydration of the RBC fosters their contribution in clot formation as outlined below. (ii) Local membrane deformation of RBCs was shown to trigger a transient increase in Ca^2+^ permeability with secondary activation of the Gardos channels [82]. This was proposed to induce significant dehydration even during a brief deformation event in the microcirculation [82].

### 3.5. Ca^2+^ and Lipid Bilayer

Scramblase is a protein responsible for bidirectional transmembrane movement of phospholipids [94] leading to the break-down of the originally asymmetrical distribution of phospholipids between the inner and outer membrane leaflet [95]. It is a passive transport, but Ca^2+^ activated [51,52]. Its Ca^2+^ sensitivity is mediated by an EF hand motif [96]. The scramblase activity is complemented by the flippase (aminophospholipid translocase) inhibition [97]. This protein actively builds up phospholipid asymmetry and such can be regarded as the opponent of the scramblase. As shown in Figure 1, flippase activity is almost completely suppressed by 400 nM Ca^2+^ [53].

### 3.6. Ca^2+^ and Metabolism

Numerous reports emphasize the possible role of the Ca-CaM system in regulation of activity of glycolytic enzymes including pyruvate kinase [98,99]. However, even more important is its pivotal role in assembling the glycolytic enzymes at the RBC membrane. Band 3 protein and its cytosolic domain was shown to serve as a docking station for multiple glycolytic enzymes [100]. Ca^2+^ in turn was suggested to promote band 3 tyrosine phosphorylation [101]. Phosphorylation of the cytosolic domain of band 3 protein (cdb3) at Tyr9 and Tyr21 results in displacement of LDH, PK, GAPDH, PFK and aldolase from RBC membrane in intact cells [102]. A similar effect is induced by interaction of deoxyHb with band 3 protein [103].

### 3.7. Ca^2+^ and Redox State Preservation

In RBCs there is a direct link between the intracellular free Ca^2+^ concentration and the haemoglobin oxygen saturation. In cells of healthy individuals, passive Ca^2+^ uptake was unaffected by deoxygenation, but the *V*_max_ of the PMCA was reduced by 18%–32% [104]. This is not the case in RBCs of patients with SCD [4]. An increase in free Ca^2+^ levels was mainly attributed to changes in haemoglobin protonation, increases in protonation of deoxyhaemoglobin and a shift in the intracellular pH towards more alkaline values [104,105]. Along with augmentation of 2,3-diphosphoglycerate and ATP binding to haemoglobin, interaction of deoxyhaemoglobin with protons is associated with a decrease in Ca^2+^ buffering capacity of haemoglobin. In the cytosol of deoxygenated RBCs release of Ca^2+^ ions from protein binding sites and lowering of extrusion capacity of the PMCA contribute to both an increase in the ionised Ca^2+^ fraction by 34%–74% even in the absence of Ca^2+^ influx from the extracellular medium [104].

Increases in the free Ca^2+^ were recently linked to a lower oxygen affinity of haemoglobin promoting the release of oxygen [33]. Deoxygenation induced transient release of Ca^2+^ from intracellular buffers may promote further deoxygenation enhancing O_2_ dissociation from haemoglobin. Molecular mechanisms of this phenomenon remain to be investigated.

Aside to the control of haemoglobin oxygenation, Ca^2+^ is also involved in the regulation of the RBC’s redox state. Ca-CaM complexes are co-activators of endothelial NO synthase (eNOS) activity [106,107]. Recently eNOS was shown to be present in circulating RBCs [108,109] and is activated by Ca^2+^ uptake during shear stress [82,110]. Nitric oxide is a scavenger of superoxide anions, which interacts with them two orders of magnitude faster than superoxide dismutase (SOD) [111]. However, following shortage of tetrahydrobiopterin or l-arginine eNOS itself gets uncoupled and is capable of generating superoxide anions, which is turned into H_2_O_2_ in a reaction catalysed by SOD [106]. Pro-oxidative action of uncoupled eNOS was demonstrated in RBCs [112].

### 3.8. Calpain and Its Targets in RBCs

While the Ca^2+^ dependent cysteine protease μ-calpain (calpain-1) was mainly detected in human RBCs, m-calpain (calpain-2) was found to be virtually absent [113]. μ-Calpain is highly sensitive to Ca^2+^ with a half-activation concentration in the range of 3–50 μM [114,115]. The enzyme isolated from human RBCs displayed a value of 40 μM [54]. This is well above the free Ca^2+^ concentration found in both human RBCs from healthy individuals (30–60 nM) and values of 100–300 nM reported in RBCs from patients with hereditary forms of anaemia [4]. However, a 40 kDa activator protein which makes μ-calpain more Ca^2+^ sensitive, shifts Ca^2+^ concentrations required for half-maximal activation from 40–50 μM down to 0.2 μM [55]. In human RBCs a fraction of membrane-associated active μ-calpain was detected [116,117]. Activation mediated recruitment of calpain to the membrane [118] was used to estimate the protease activity revealing that ~7% of the calpain pool is constitutively active in RBCs from healthy individuals [117]. Consequently, the μ-calpain activity in RBCs from patients with hereditary anaemia forms was also increased because their resting Ca^2+^ levels were raised when compared to those from healthy donors. [4].

Targets of activated calpain are mainly transmembrane or membrane-associated proteins including PMCA, and bands 1, 2, 2.1, 3, 4.1, 4.2 proteins, but also calpain itself [119]. Autolysis occurs at Ca^2+^ levels beyond physiological concentrations, namely in the range 50–150 μM [114]. Limited digestion of haemoglobin α and β chains by calpain was also reported [120].

Recently, RBCs of a μ-calpain knock-out (KO) mouse displayed an improved deformability [121]. Furthermore it was demonstrated that ankyrin, band 3, band 4.1R, adducin and dematin were degraded in the Ca^2+^ loaded normal RBCs but not in the KO RBCs [121].

Cleavage of the PMCA by μ-calpain was associated with an activation of the pump, rapid ATP depletion, inactivation of the pump and gradual loss of the transmembrane Ca^2+^ gradients [122].

Calpastatin, an endogenous inhibitor of μ-calpain, is a natural regulator of the enzyme activity in RBCs. Both major and minor components of calpastatin, calpastatin H and L were detected in human RBCs [54]. Interaction of calpastatin with calpain is also Ca^2+^ sensitive. Half-maximal activation of calpastatin occurs at 40 μM Ca^2+^ [114], in bovine skeletal muscle derived enzyme. Activity of calpain and a decrease in calpastatin levels in RBCs was shown to occur in elderly humans [123,124].

### 3.9. Ca^2+^ and Inter-Cellular Interactions

Due to the protein activity and lipid remodelling described above one would expect changes in rheological properties of RBC suspensions and indeed in numerous studies provoking increased cytosolic Ca^2+^ levels, an altered rheology was observed [125–127]. These changes were majorly explained by altered RBC deformability [128,129]. However, a second possibility to explain changes in rheological properties of RBC suspensions is RBC aggregation.

A participation of RBC Ca^2+^ channels in blood clot formation was proposed previously [92,93] following earlier work already suggesting an active participation of RBCs in blood clot formation [130–132]. Further support was given by investigations showing Ca^2+^ mediated aggregation of RBCs [133–136] and RBC adhesion to the endothelium [137–139]. Further investigations are necessary to specify the required and sufficient conditions for such processes, e.g., the threshold of the intracellular Ca^2+^ concentration at which aggregation occurs. Additionally the modulations of the aggregation properties under *in vivo* conditions need to be elucidated.

## 4. The Physiological Role of Intracellular Ca^2+^: From RBC Birth to Clearance

### 4.1. Ca^2+^ in RBC Haematopoiesis

Ca^2+^ uptake is of key importance for promoting differentiation and proliferation of erythroid precursors at the stages of burst-forming units erythroid (BFU-E) colony-forming units erythroid (CFU-E) [140,141]. Increase in the intracellular Ca^2+^ is an integral part of the signalling pathway activated by binding of erythropoietin to its receptor [142,143]. In Ca^2+^-free medium, Ca^2+^ uptake is absent and differentiation and survival of erythroid precursors is compromised [140,141]. Inhibition of Ca^2+^ uptake by erythroid precursor cells cultured from mononuclear cells by the NMDA receptor antagonist MK-801 resulted in 45.5% ± 12.8% mortality of cells at the stage of basophilic and polychromatic erythroblasts suggesting that these receptors are actively contributing to erythropoiesis [33]. Additional evidence indicating that Ca^2+^ levels in reticulocytes are higher than in mature RBCs comes from secondary Ca^2+^-dependent processes such as phosphorylation [144]. Protein 4.1R phosphorylation by PKC appeared to be markedly elevated in reticulocytes resulting in weakened interaction between β-spectrin and actin [145].

### 4.2. Ca^2+^ in Relation to the Physiological Function of RBCs

For a long time, the physiological function of Ca^2+^ in mature RBCs was obscure and was believed to be limited to the involvement in RBCs aging and clearance [10,146,147]. However, a prominent part of this report reviews the physiological functions of Ca^2+^ in RBC regulating a broad range of processes including O_2_ transport [33], rheology [148], clotting [135,136] and half-life of cells (see Section 4.3). Each of these functions is vital for the organism. Thus, aberrant Ca^2+^ homeostasis in RBCs results in development of severe life-threatening systemic pathologies.

Very recently, additional evidence in favour of a physiologically important Ca^2+^ associated mechanism was reported. Here, rises in the intracellular Ca^2+^ appear to promote the ability of RBCs to deliver oxygen [33].

### 4.3. Ca^2+^ in RBC Clearance

At present it is suggested that in senescent RBCs the intracellular Ca^2+^ levels exceed those in reticulocytes and young RBCs [149]. However, such conclusions on the relationship between cell age and steady-state Ca^2+^ levels largely depend on the age markers employed. Typical age markers include glycosylated haemoglobin HbA1c, band 4.1a/b ratio, cell density, de-sialation and changes in CD47 abundance at the membrane surface, PS exposure, and several others [149–151].

Activity of the PMCA in RBCs was shown to decrease with progressing HbA1c accumulation [152]. However, based on the pump-leak theory, this process will result in Ca^2+^ accumulation only when coupled to the unchanged or increasing activity in Ca^2+^-transporting ion channels. However, according to recent findings, this is not necessarily the case. Young rat and human RBCs contain higher number of NMDA receptors, that upon stimulation with plasma glycine and glutamate can cause significant Ca^2+^ influx [32,33]. Young cells are preferentially removed in subjects with induced or chronic polycytemia, phenomenon known as neocytolysis [112,153–155]. Finally, phosphatydylserine (PS) exposure does not always correlate with high Ca^2+^ levels [156]. Thus, both young and senescent RBCs appear to be prone to Ca^2+^ overload, which may well trigger RBC clearance, but the relation of this mechanism to other Ca^2+^ independent clearance mechanisms and to *in vivo* relevance is still obscure.

## 5. Ca^2+^ Dysbalance and Haemolytic Anaemia

Independent of its origin, hereditary haemolytic anaemia is often associated with an increase in the intracellular Ca^2+^ levels [4]. “Leakiness” of RBC plasma membrane for Ca^2+^ that could not be compensated for by the activation of PMCA was reported for patients with SCD [157–159], beta-thalassemia (although most of the Ca^2+^ seem to be sequestered in vesicles or bound to cytosolic proteins) [160–162], phosphofructokinase deficiency [163]. Most of the information on Ca^2+^ transport in diseased RBCs was so far obtained for SCD patients. In these cells relatively high rates of Ca^2+^ uptake in RBCs are partially compensated for by sequestration of Ca^2+^ into intracellular inside-out vesicles, in which Ca^2+^ is pumped actively by PMCA [164]. Apparently, this process also exists in RBCs of patients with β-thalalssemia intermedia [162,165]. Furthermore, part of Ca^2+^ taken into the cells is immobilised by the intracellular proteins. Sickle cell transformation associated with polymerisation of deoxygenated mutated *S*-haemoglobin is amplified by 20–40-fold by dehydration [166]. Deoxygenation promotes Ca^2+^ uptake and release of ionised Ca^2+^ from the intracellular proteins (reduction in buffering capacity) [104,105,167]. In deoxygenated SCD RBC, an acute increase in the intracellular free Ca^2+^ RBCs causes opening of the Ca^2+^ sensitive Gardos channel and anion channels [168–170].

Downstream events triggered by augmentation of free intracellular Ca^2+^ comprise activation of μ-calpain [171] and activation of tyrosine phosphorylation [172]. In RBCs from SCD patients, protein 4.1R and p55 appear to be phosphorylated thus contributing to the weakened interaction with beta-spectrin [84].

Cross-linked polymers have been observed in RBCs of patients with SCD suggesting hyperactivation of transglutaminase [85].

Oxidation of membrane proteins and impaired NO production by eNOS [173,174], increase in intercellular and RBC-endothelial adhesion [175] are hallmarks of SCD. Cross-linking of the nature of carrier of sickle cell conductance (*P_sickle_*) mediating Ca^2+^ uptake by RBCs remains unknown (compare Section 2.2).

## 6. RBC Ca^2+^ Content and Medicinal Side Effects

### 6.1. Transfusion Medicine

Ca^2+^ overload is involved in dramatic reduction of the life span of stored RBCs used for transfusion [176]. Storage of leuco-depleted RBC concentrates is currently performed in Ca^2+^ free glucose-containing preservation citrate buffers at low temperatures. These storage conditions favour Ca^2+^ depletion of the cytosol, oxidative stress and ATP deprivation [177,178]. These processes induce deactivation of PMCA, facilitate passive Ca^2+^ transport and evoke acute Ca^2+^ overload when re-exposed to the plasma of patients receiving transfusions. The resulting processes include dehydration, rigidity, fragmentation of cytoskeletal proteins and oxidative stress and increased adherence of RBCs to the endothelium and to each other [179,180].

### 6.2. Therapeutic Side Effects

Additionally, unwanted modulations of the Ca^2+^ entry into RBC may cause side effects of drugs involved in therapies unrelated to RBCs. An example is photodynamic therapy, where the oxidative stress produced by the photosensitizer leads to the activation of cation channels in the RBC membrane and the consecutive Ca^2+^ entry triggers the mechanism described above, which is the major cause of an increased formation of blood aggregates as well as haemolysis [181]. Thus, RBC remain to be model cells to develop pharmacological strategies [182] and can even be used in automated safety screens [183].

## 7. Conclusion and Perspective

The role of Ca^2+^ in RBCs physiology and pathophysiology cannot be overestimated. Many links between Ca^2+^ and RBC related diseases still need to be explored [184]. Methodologically single cell based methods will increase in their importance and contribution and complement cell population measurements [185]. This is due to the recent hindsight that intercellular heterogeneity and, in some cases, inhomogeneous distribution of Ca^2+^ within the cytosol are essential to predict the onset of changes related to the abnormally high Ca^2+^ levels, which are particularly important in patients with haematological disorders [42,186]. However, the properties of Ca^2+^ binding entities within the cells will need further attention and research. Following the broad variety of Ca^2+^ mediated processes mentioned here, monitoring the following parameters may be used to indirectly predict abnormally increased intracellular free Ca^2+^ levels in RBCs: (i) changes in cell volume and morphology (microcytosis, high MCHC, increase in cell density, echinocytosis or stomatocytosis); (ii) congenital haemolytic anaemia associated with stomatocytosis, reticulocytosis, and shortened RBC survival; (iii) decrease in the intracellular K^+^ levels, pseudohyperkalemia; (iv) loss of RBC deformability, changes in osmotic resistance, an increase when dehydration has occurred but cytoskeletal stability is still maintained, or a decrease when cytoskeleton is partially disassembled; (v) appearance of calpain-induced band 3 cleavage fragments; (vi) oxidative stress or unusually high NO production (nitrosated Hb, met-Hb); (vii) ATP depletion due to hyperactivation of PMCA; (viii) increase in inter-RBC aggregability; and (ix) increase in PS exposure.

For many years the RBC was the cell of choice for membrane transport investigations. In the age of genomics, interest in RBC research decreased, but numerous signalling cascades—also in respect to the second messenger Ca^2+^ that occur in other cells and may involve several organelles—have been rediscovered in a modified and/or simplified form in RBCs.
